# Numerical analysis of blood flow in the abdominal aorta under simulated weightlessness and earth conditions

**DOI:** 10.1038/s41598-024-66961-7

**Published:** 2024-07-10

**Authors:** Marta Żyłka, Grzegorz Górski, Wojciech Żyłka, Agnieszka Gala-Błądzińska

**Affiliations:** 1 The Faculty of Mechanical Engineering and Aeronautics, Department of Aerospace Engineering, Rzeszow University of Technology, av. Powstańców Warszawy 8, 35-959 Rzeszów, Poland; 2 Institute of Physics, College of Natural Sciences, University of Rzeszów, ul. Pigonia 1, 35-310 Rzeszów, Poland; 3 Institute of Materials Engineering, College of Natural Sciences, University of Rzeszów, ul. Pigonia 1, 35-310 Rzeszów, Poland; 4 Institute of Medical Sciences, Medical College of Rzeszow University, Al. mjr. W. Kopisto 2a, 35-959 Rzeszów, Poland; 5 Internal Medicine, Nephrology and Endocrinology Clinic, St. Queen Jadwiga Clinical District Hospital No. 2 in Rzeszow, ul. Lwowska 60, 35-301 Rzeszów, Poland

**Keywords:** Biomedical engineering, Computational science

## Abstract

Blood flow through the abdominal aorta and iliac arteries is a crucial area of research in hemodynamics and cardiovascular diseases. To get in to the problem, this study presents detailed analyses of blood flow through the abdominal aorta, together with left and right iliac arteries, under Earth gravity and weightless conditions, both at the rest stage, and during physical activity. The analysis were conducted using ANSYS Fluent software. The results indicate, that there is significantly less variation in blood flow velocity under weightless conditions, compared to measurement taken under Earth Gravity conditions. Study presents, that the maximum and minimum blood flow velocities decrease and increase, respectively, under weightless conditions. Our model for the left iliac artery revealed higher blood flow velocities during the peak of the systolic phase (systole) and lower velocities during the early diastolic phase (diastole). Furthermore, we analyzed the shear stress of the vessel wall and the mean shear stress over time. Additionally, the distribution of oscillatory shear rate, commonly used in hemodynamic analyses, was examined to assess the effects of blood flow on the blood vessels. Countermeasures to mitigate the negative effects of weightlessness on astronauts health are discussed, including exercises performed on the equipment aboard the space station. These exercises aim to maintain optimal blood flow, prevent the formation of atherosclerotic plaques, and reduce the risk of cardiovascular complications.

## Introduction

### Physiological changes in the body while on the space station

During the stay at a space station, an astronaut is exposed to changes in physiological processes and psychosomatic changes in the body, resulting from prolonged exposure to gravitational inertia. This can lead to decrease the strength^1^ in muscle mass, decrease bone density—followed by loss of bone mass and finally to osteopenia and osteoporosis^2^.

A prolonged stay at the space station impairs balance, motor coordination, and proprioception^3^. Changes in the cardiovascular system, such as a decrease in total body water and intravascular body water volume, increased risk of thrombosis and embolism, and decrease in cardiac performance, can occur in weightless environments^4,5^. Additionally, weightlessness causes hormonal homeostasis disorders such as decreased testosterone levels in men^6^. Additionally, disorders of the gut microflora can affect gastrointestinal function^7^, including sleep problems^8^. Moreover, psychological changes, such as feelings of social isolation, decreased motivation and mood, and an increased risk of psychiatric disorders, such as depression and anxiety, are also associated risks^9–11^.

### Countermeasures used at the space station

Maintaining health and fitness during long-term space missions is critical for astronauts^12,13^. Hence, extensive research has led to the development of drugs, that mitigate the harmful physiological, structural, and psychological effects of prolonged weightlessness^14^. These drugs have been used since 1973 and continuously improved over the years. As part of these continued efforts, new devices have been designed, and existing devices have been improved to optimize physical training and alleviate cardiovascular problems, osteoporotic bone changes, and skeletal muscle atrophy.

One of the first training devices introduced at the International Space Station (ISS) was the Interim Resistance Exercise Device (iRED)^15,16^, which is used to perform resistance exercises under microgravity conditions. However, no resistance exercises were performed using the device, since it was incapable of inducing loads comparable to those of exercises performed on the Earth. Consequently, maintaining normal muscle and bone mass during a long-term stay in space was difficult^17^.

Subsequently, an Advanced Resistance Exercise Device (ARED) was introduced in the ISS^18^. It uses piston-driven vacuum cylinders with adjustable resistance in combination with a flywheel system to replicate free-motion exercises. Notably, this device accommodates the varying anthropometric parameters of astronauts. Astronauts can perform exercises using a lifting bar or rope set^19^, enabling resistance exercises to activate all muscle groups^20^.

Performing aerobic exercises, such as running on a T2 treadmill^21^, is important for maintaining the physical condition of astronauts as it allows them to maintain muscle strength and heart and respiratory system efficiency. T2, equipped with harnesses and elastic straps, simulates body weight and allows astronauts to run or walk on a moving belt. These exercises combine workload and high-intensity elements^22^.

A treadmill with a vibration isolation system (TVIS) is an advanced device designed to minimize the effects of gravity on the body of astronauts during exercise, allowing them to maintain their health and fitness during long stays in space^23^. The use of this device in space stations allows astronauts to minimize the negative effects of microgravity on the body, such as bone loss and loss of muscle mass^24,25^.

Another type of ergometer used on the ISS for astronauts’ physical exercise is the Cycle Ergometer with Vibration Isolation (CEVIS), an advanced exercise bike^25,26^. The device does not require a saddle for weightlessness and is primarily used for cardiovascular training^27^. Astronauts mounted bicycles to the floor on the ISS, and strapped their feet to the pedals to move their lower limbs, while also being strapped to the device to maintain correct position^28^.

The Colbert treadmill is an advanced exercise device used at the space station to keep astronauts healthy and fit during long-term space missions^29,30^. The device has two modes of operation: active powered mode, wherein the treadmill is powered by an electric motor, and passive powered mode, wherein the runner presses his/her lower extremities against a moving treadmill belt^31^.

Systemic vibration exercise (RVE) is an innovative approach to physical training used in the space station to address microgravity musculoskeletal dysfunction^32^. Clinical studies have been conducted to confirm the effects of systemic vibration exercises on bone health during spaceflight^33^. This type of training is effective in preventing bone and muscle atrophy^34,35^ and is recommended not only during space missions but also before and after space travel to maintain astronauts’ health. Stimulation provides mechanical and neural signals, that positively affect neuromuscular connections^26^.

### Blood flow in a state of weightlessness

In a state of weightlessness, hemodynamic conditions change in the body can affect cardiovascular function^36,37^. In the weightless state, blood undergoes rheological changes and an increase in viscosity^38^ affecting the resistance to flow and the dynamic properties of the blood flow^39^. Furthermore, changes in posture and the absence of gravitational forces increase blood volume in the upper parts of the body and decrease blood volume in the lower parts^40–42^. This can affect cardiovascular function, increase the risk of thrombosis, and adversely affect heart function^43–45^.

With appropriate preventive measures, such as: physical training during spaceflight and fluid resuscitation upon return no adverse effects have been observed. A study conducted by Fu et al.^46^ on 12 astronauts showed that after six months in space, no astronauts experienced orthostatic hypotension during daily activities in the first 24 h after landing. Although prolonged spaceflight exposure slightly affected the variability in systolic blood pressure, this was only a transient spatial change associated with mild hypotension. All these values returned to normal after returning to Earth^46^.

The effect of weightlessness on blood flow in the human body remains under investigation and requires further research to fully understand the underlying mechanisms. However, testing body functions invasively by assessing intravascular blood flow under microgravity would be unethical. Therefore, the conditions were simulated without astronauts using computational fluid dynamics (CFD). CFD can provide doctors with valuable decision-making support for the treatment of aortic diseases. It enables the creation of modern individualized diagnostic and therapeutic plans. Consequently, doctors have better tools to develop individualized treatment plans for patients with aortic disease, contributing to improving medical care in this region^47–49^. Simulating cardiovascular function under microgravity can provide valuable information for monitoring astronaut health.

In this study, blood flow through the abdominal aorta and left and right iliac arteries was simulated under the influence of Earth’s gravity in a weightless state at rest and during exercise. The ANSYS 2023 Fluent software was used for simulations using the standard k-ω turbulent flow model. ANSYS is a versatile software that allows analyses in areas such as structural mechanics^50–52^, heat transfer, magnetic and electric field, and fluid mechanics^53,54^. It also solves mixed problems by combining the above systems, including fluid–solid interactions (FSI).

The aorta and artery model was built based on the results of an angiography study. A computed tomography scan (CT) of the aorta and iliac arteries was obtained courtesy of the Internal Medicine Department of the provincial hospital, along with archival documents of a deceased patient (death due to infectious reasons), without disclosing their name and other data that could allow identification. Angio-CT of the aorta and iliac arteries was performed using spiral acquisition, 0.6 mm slices with ECG gating (section of the thoracic aorta), after intravenous administration of a contrast agent in a 39-year-old man admitted to the hospital, because of syncope during the course of reactive hypoglycemia. The patient had simple obesity (BMI = 35 kg/m^2^) without other chronic diseases, including cardiovascular diseases.

## Model

### Simulation assumptions

**Figure 1:**
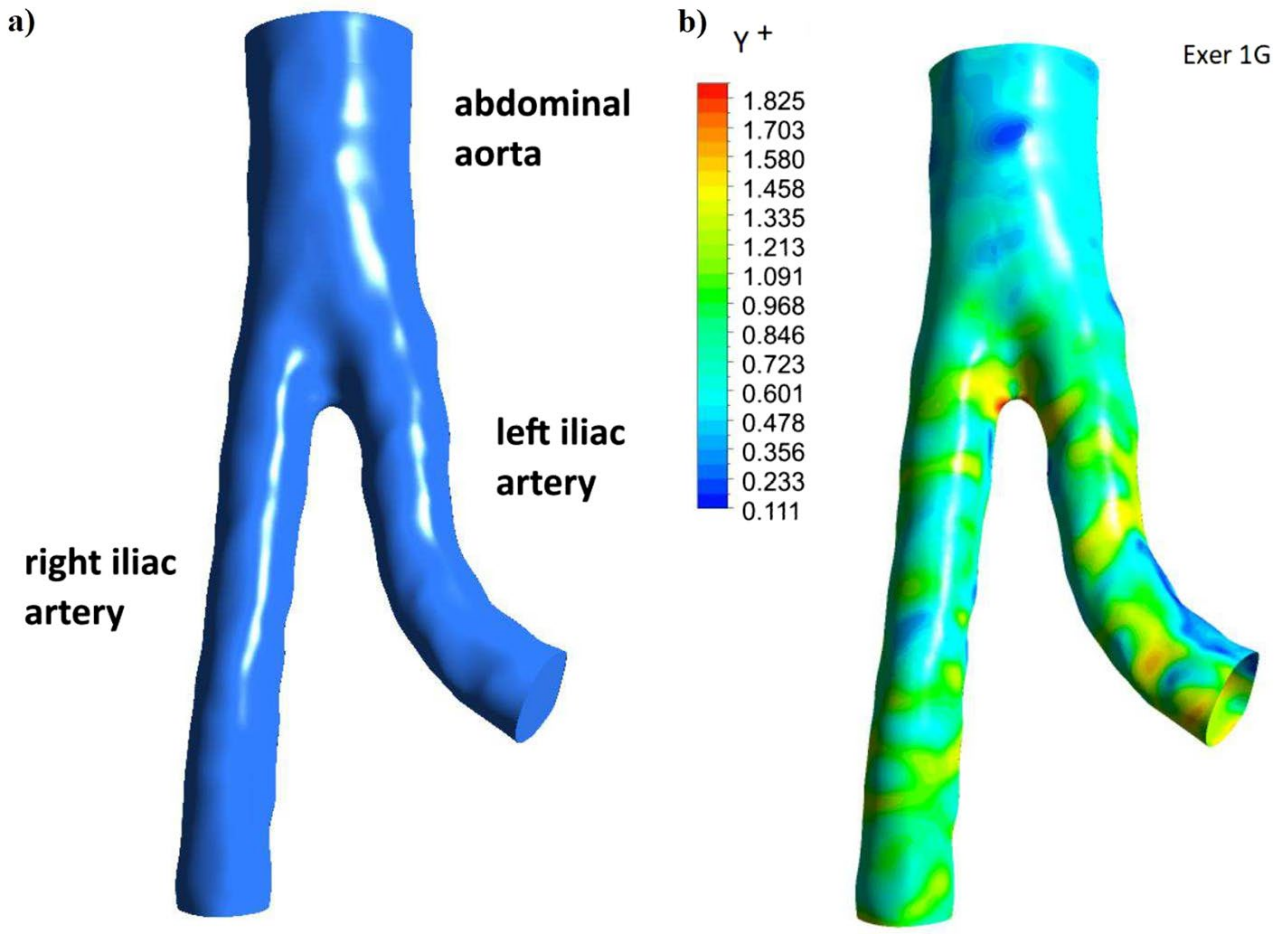
(**a**) Model of abdominal aorta and iliac arteries, and (**b**) wall Y^+^ for systolic pressure during moderate exercise and earth gravity.

**Figure 2:**
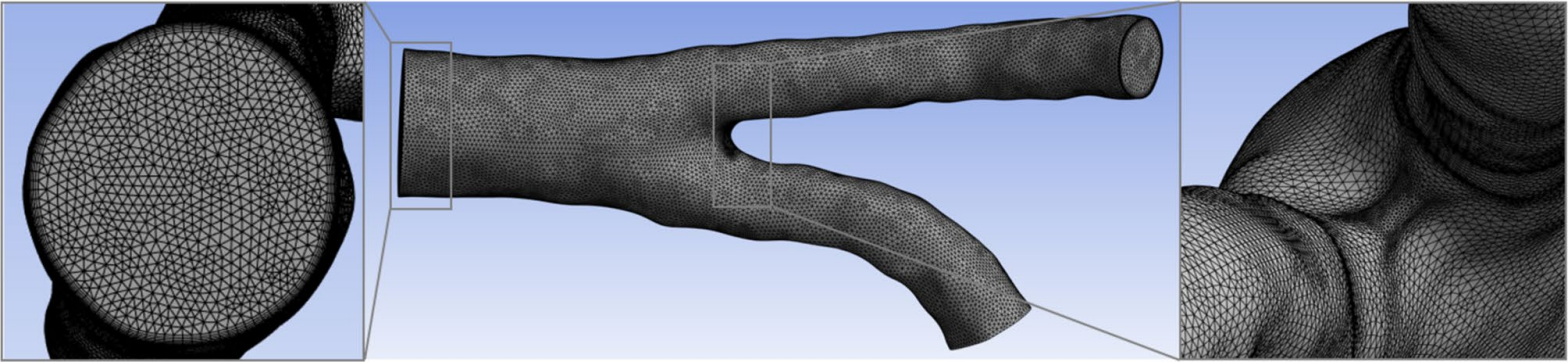
Computational grid of the object under study, detailing the layers of inflation.

Numerical analysis was performed using ANSYS 2023R1 Fluent software. We considered blood flow through the abdominal aorta (AA), left iliac arteries (LIA), and right iliac arteries (RIA) (see Fig. 1a). The 3D geometries of the abdominal aorta and iliac arteries were prepared in the Ansys SpaceClaim environment from CT images processed using a 3D Slicer. The inlet aorta diameter was 16 mm. The generated mesh is shown in Fig. 2.

The geometry shown in Fig. 2 consisted of 994,726 tetrahedral elements with seven layers inflated at the wall site. For simulations using the Reynolds-averaged Navier–Stokes (RANS) model, preparation good mesh is important, especially in areas, where the flow direction changes rapidly. In our model, this rapid change occurs for the aortic bifurcation, so we additionally refined the computational mesh in this area. This additional density is visible in the right side of Fig. 2.

The selection of the element size was preceded by a mesh-independent study. In Table 1, we present a comparison of the maximum velocity $(u_{max} )$, maximum values of wall shear stress $(WSS_{max} )$ and mean values of WSS for four meshes. Initial conditions for exercise and Earth’s gravity were applied for the calculations. The values of $u_{max}$ and $WSS_{mean}$ mean are very similar. For these units, we ensure $y^{ + } < 1.89$ using the pressure obtained during controlled exercise and Earth’s gravity. The largest wall area is $y^{ + } < 1$, as illustrated in Fig. 1b.

**Table 1:** Mesh independent study.

Mesh	Number of elements	$u_{max}$[m/s]	$WSS_{max}$[Pa]	$WSS_{mean}$[Pa]
Coarse	532,453	1.95	93.92	14.74
Medium	783,478	1.96	91.45	14.77
Fine	994,726	1.95	91.82	14.77
Extra fine	1,671,045	1.94	96.40	14.78

For the analysis, blood was assumed to be incompressible, with a density $\rho = 1060\;{\text{ kg/m}}^{2}$, and a dynamic viscosity of the blood *μ* = 0.0035 Pa·s^55^. The maximum average input velocity for the systolic peak was 0.816 m/s for exercise and 0.533 m/s for rest, resulting in Reynolds numbers $Re = \frac{\rho ud}{\mu }$ of 3954 and 2582, respectively.

In the environment of zero gravity, the average input velocity was 0.684 m/s for exercise and 0.402 m/s for rest, corresponding to Reynolds numbers Re = 3313 and Re = 1948. The resulting Re number was between the lower and upper critical Reynolds numbers. Hence, the flow was classified as transient, and the SST $k - \omega$ model was used for the analysis. This model is often used to analyze the transient blood flow^56–60^. As reported by Banks and Bressloff^58^, the $k - \omega$ arterial flow model is more consistent with the experimental results for large arteries. For the case involving gravity, we used the typical Earth gravitational acceleration of 9.81 m/s^2^.

### Mathematical models

A mathematical model can be used to describe any physical process involving fluid flow. When performing numerical simulations, selecting an appropriate model that closely mimics the real-world phenomena is critical. The Navier–Stokes equations are commonly used for this purpose, comprising mass conservation (1) and momentum conservation (2) equations^53,54,61^.

Mass conservation equation:

1 $$\frac{\partial \rho }{{\partial t}} + \frac{\partial }{{\partial x_{j} }}\left( {\rho u_{j} } \right) = 0$$

Momentum conservation equation:

2 $$\frac{\partial }{\partial t}\left( {\rho u_{i} } \right) + \frac{\partial }{{\partial x_{j} }}\left( {\rho u_{j} u_{i} } \right) = \rho f_{i} - \frac{\partial p}{{\partial x_{i} }} + \frac{\partial }{{\partial x_{i} }}\left[ {\mu \left( {\frac{{\partial u_{i} }}{{\partial x_{j} }} + \frac{{\partial u_{j} }}{{\partial x_{i} }}} \right)} \right] + \frac{{\partial \tau_{ij} }}{{\partial x_{j} }}$$

where: $x_{i} = \left( {x,\;y,\;z} \right)$ represents positions in the Cartesian coordinate systems, $u_{i}$—mean velocity component in the $x_{i}$ direction, $\rho$—fluid density, $f_{i}$—body accelerations component, *p*—static pressure, $\mu$—represents fluid dynamic viscosity and $\tau_{ij}$—the Reynolds stress tensor.

In our simulation, the body acceleration vector is equal to $\vec{f}$ = [0, 0, − 9.81] m/s^2^ for 1G case and $\vec{f}$ = [0, 0, 0] m/s^2^ for 0G case.

For incompressible flow, the continuity equation^53,62^ can be written according to the principle of mass conservation as follows:

3 $$\frac{{\partial u_{i} }}{{\partial x_{i} }} = 0$$

The equation describes the conservation of mass in flow. It indicates, that the differences of the velocity field is equal to zero, which means that the mass flux in a given area remains constant over time. The principle of momentum conservation, considering possible turbulent flows, is expressed by the averaged Navier–Stokes (RANS):

4 $$\frac{{\partial u_{i} }}{\partial t} + \frac{\partial }{{\partial x_{j} }}(u_{j} u_{i} ) = f_{i} - \frac{1}{\rho }\frac{\partial p}{{\partial x_{i} }} + \frac{1}{\rho }\frac{\partial }{{\partial x_{j} }}\left[ {\mu \left( {\frac{{\partial u_{i} }}{{\partial x_{j} }} + \frac{{\partial u_{j} }}{{\partial x_{i} }}} \right)} \right] + \frac{1}{\rho }\frac{{\partial \tau_{ij} }}{{\partial x_{j} }}$$

This equation describes the momentum behaviour of fluid in flow. It takes into account the influence of changes in pressure, density, viscosity, and turbulent stresses on fluid motion. Reynolds stress tensor is given as follows:

5 $$\tau_{ij} = - \rho \left\langle {u^{\prime}_{i} u^{\prime}_{j} } \right\rangle \ge \mu_{t} \left( {\frac{{\partial u_{i} }}{{\partial x_{j} }} + \frac{{\partial u_{j} }}{{\partial x_{i} }}} \right) - \frac{2}{3}k\delta_{ij}$$

where $u^{\prime}_{i}$ is the fluctuating velocity component, $\mu_{t}$ is the turbulent eddy viscosity and $k = \frac{1}{2}\left\langle {u^{\prime}_{i} u^{\prime}_{j} } \right\rangle$ denotes the turbulent kinetic energy. The Reynolds stress tensor consists of three components: turbulent production stress, turbulence viscosity, and a component related to turbulent kinetic energy. Using the standard $k - \omega$ model^63,64^, the turbulent viscosity is computed from:

6 $$\mu_{t} = \frac{\rho k}{\omega }$$

where $\omega$ is the specific dissipation rate. The turbulent viscosity is calculated based on the turbulent kinetic energy *k* and the dissipation rate coefficient *ω*. This value is crucial for modelling turbulent fluid flow.

### Boundary conditions

**Figure 3:**
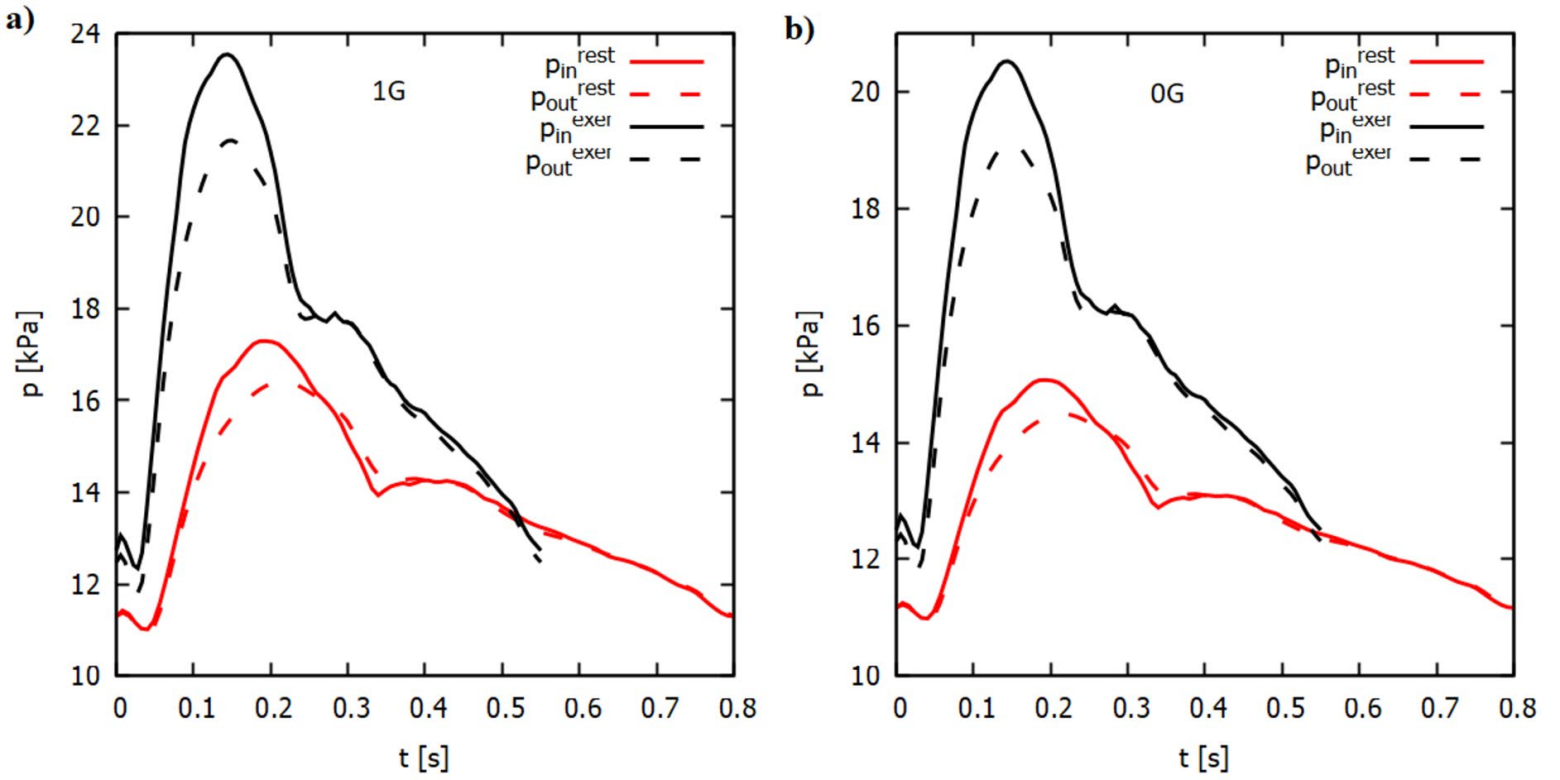
Waveform of pressure inlet (in) and outlet (out) at rest (red) and with moderate exercise (black) for the 1G case (**a**) and the 0G case (**b**).

Different boundary conditions (bc) at the inlet, for example inlet velocity bc, mass flow bc and inlet pressure bc are used in models analyzing blood flow. In our work, when analyzing blood flow in the presence of microgravity, we used information about the change in blood pressure. Therefore, for the simulation, we used the inlet dynamic pressure bc for the AA and outlet static pressure bc for the iliac arteries. The total inlet and static outlet pressures at rest and during moderate exercise are shown in Fig. 3a. The shape of the static pressure–time relationship was developed based on the work of Olufsen et al.^65^, while the course of the total inlet pressure was selected such that the intensity of blood flow at rest and during exercise was similar to the results presented by Cheng et al.^66^. The pulsation period at rest and during moderate exercise is 0.8 s (75 beats per minute) and 0.55 s (109 beats per minute), respectively. Numerical calculations were performed for two cardiac cycles and the results from the second cardiac cycle were used for analysis. For rest time step was 0.01 s and for exercise was 0.005 s. For the residuals of the continuity equation and X, Y and Z velocities, the convergence criteria were set to 10^−4^.

In microgravity, we assumed a reduction in systolic pressure. Fu et al.^46^, based on continuous finger pressure measurements, reported that during flight, the systolic pressure dropped by approximately 11.7%, while the drop in diastolic pressure was negligible. No significant changes in the heart rate were observed during flight; therefore, the pulsation period at rest and during moderate exercise could be assumed to remain unchanged.

The shape of the relationship between the total inlet and static outlet pressures, used as boundary conditions for the microgravity case (Fig. 3b), was determined as follows: the static systolic pressure for 0G ($p_{sys}^{0G}$) was equal to 88.3% of the systolic pressure for 1G ($p_{sys}^{1G}$); the diastolic pressure for 0G was equal to the diastolic pressure for 1G ($p_{dia}^{1G} = p_{dia}^{0G}$); the remaining pressures were scaled after entry

$$p_{{}}^{0G} = p_{dia}^{0G} + \frac{{p_{sys}^{0G} - p_{dia}^{0G} }}{{p_{sys}^{1G} - p_{dia}^{1G} }}\left( {p_{{}}^{1G} - p_{dia}^{1G} } \right)$$

where $p_{{}}^{0G}$ and $p_{{}}^{1G}$ denote the instantaneous pressure values for the 0G and 1G case.

The pulsation period for 1G is similar to that for 0G.

## Results

### Blood velocity

**Figure 4:**
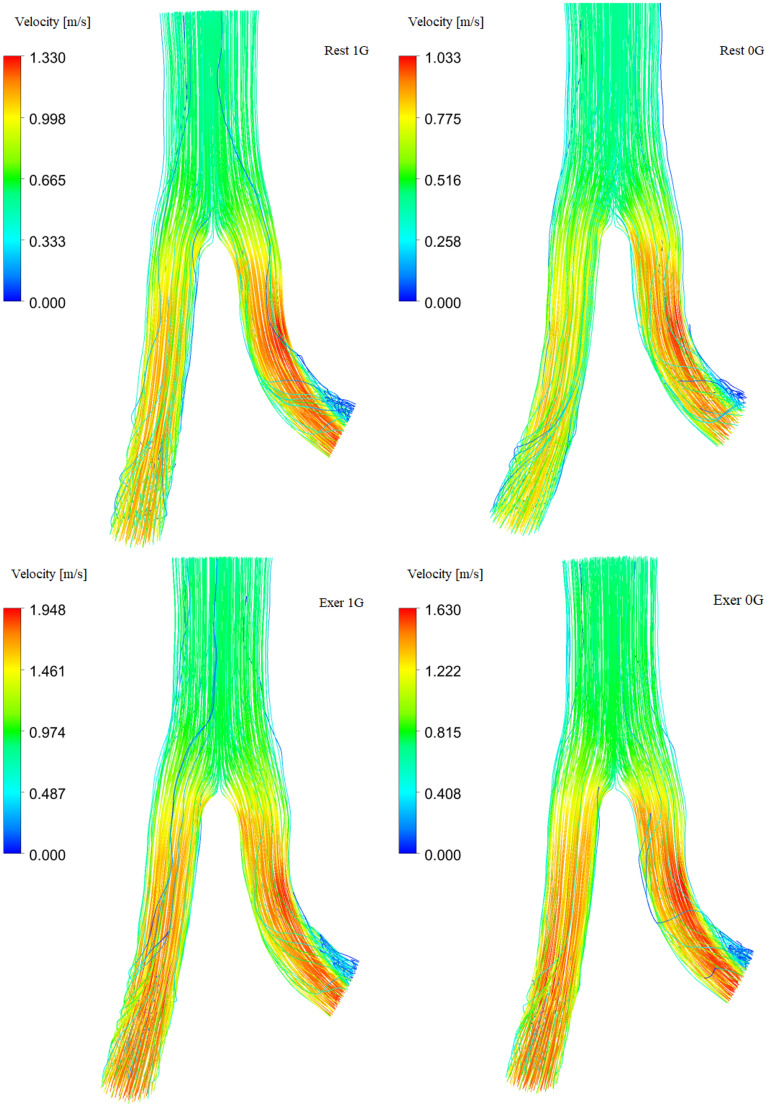
Velocity magnitude and streamline field for systolic pressure peak under 1G (left column) and 0G (right column).

We begin by discussing the results of the analysis of blood distribution flow velocity. Figure 4 shows the velocity magnitude and streamline field for the systolic pressure peak. The top row shows the results at rest and the bottom row shows the results during moderate exercise. In microgravity (right column), we observed a decrease in the maximum value by approximately 16.3% during exercise and 22.3% for the rest. The layout of the streamlines remained unchanged. Turbulence related to the large curvature of the left iliac artery was observed.

**Figure 5:**
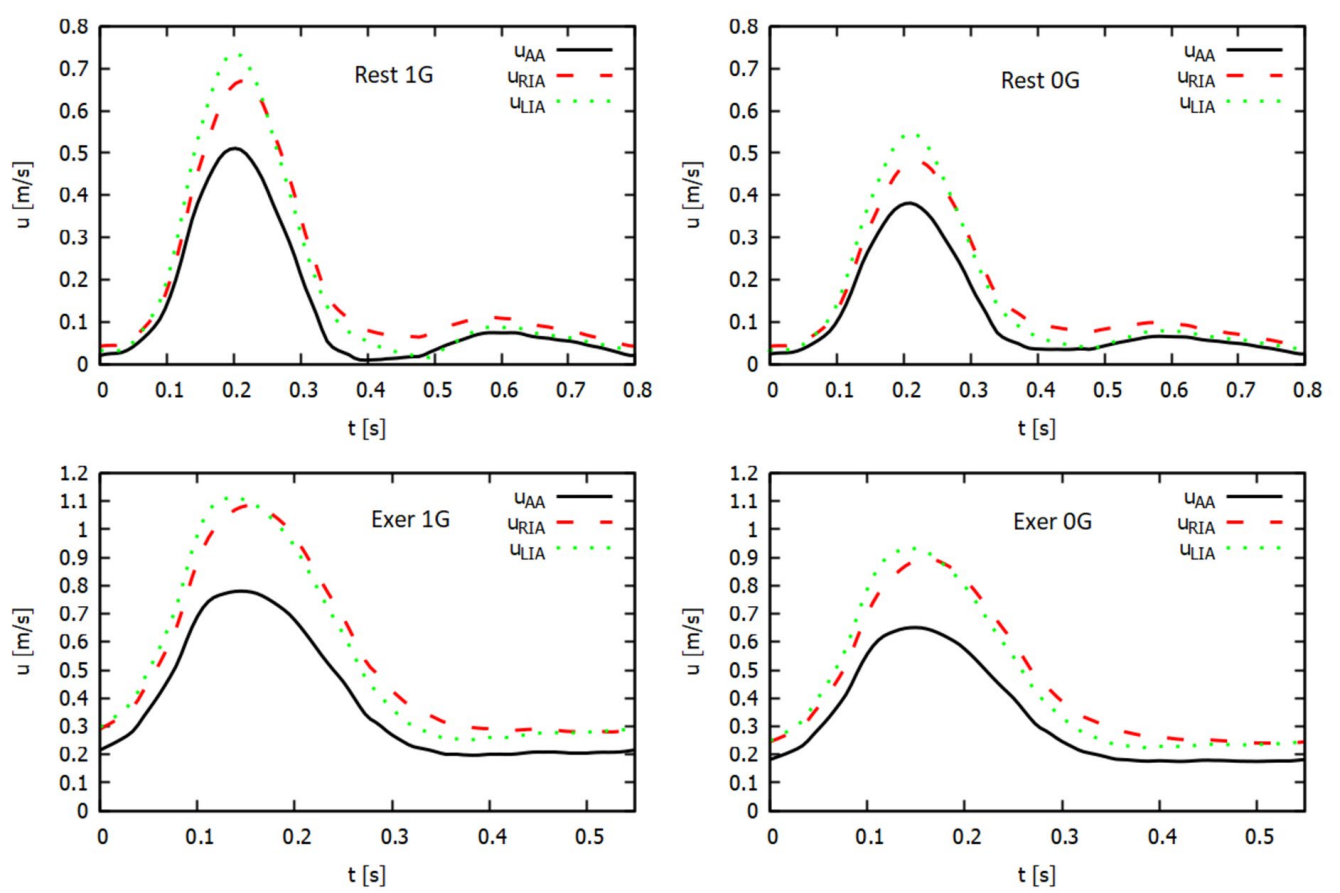
Variation in the magnitude of mean flow velocity magnitude for abdominal aorta ($u_{AA}$) and iliac arteries ($u_{RIA}$ and $u_{LIA}$) in terms of time at rest and during exercise.

In Fig. 5, we present the average velocity magnitude waves at the inlet aorta and outlet arteries. In microgravity, we observed much weaker variability in speed in Earth’s gravity; the value of the maximum speed decreased, while the minimum speed increased. For the left iliac artery, we observed higher speed values for the systole peak and lower values during early diastole.

Exercise improves blood flow speed in the abdominal aorta and iliac arteries under both terrestrial and microgravity conditions. Therefore, using preventive measures in the form of exercises on a space station is necessary so that the astronaut can maintain the blood flow velocity in the body’s large arteries similar to that occurring at rest under gravity conditions.

**Figure 6:**
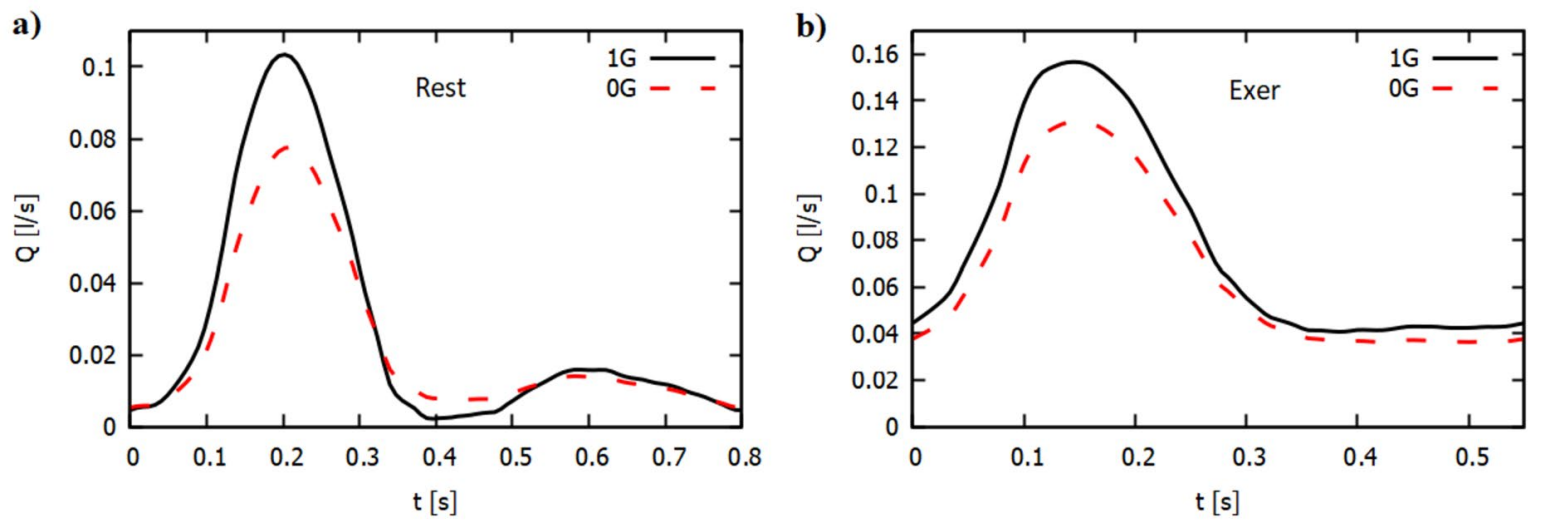
Variation of flow rate through the AA at rest (**a**) and during exercise (**b**) for 1G and 0G.

In Fig. 6, we present a comparison of the flow rate in AA, Q [l/s], for 1G and 0G at rest (Fig. 6a) and during exercise (Fig. 6b). At moderate speeds, in the presence of microgravity, we observed a strong decrease in the flow rate, especially for systolic pressure^67^. For diastolic pressure drop, the flow rate under microgravity was much lower. At rest, the average flow rate in 0G dropped by approximately 16%, whereas during exercise, the decrease was approximately 14.8%.

### Wall shear stress analysis

**Figure 7:**
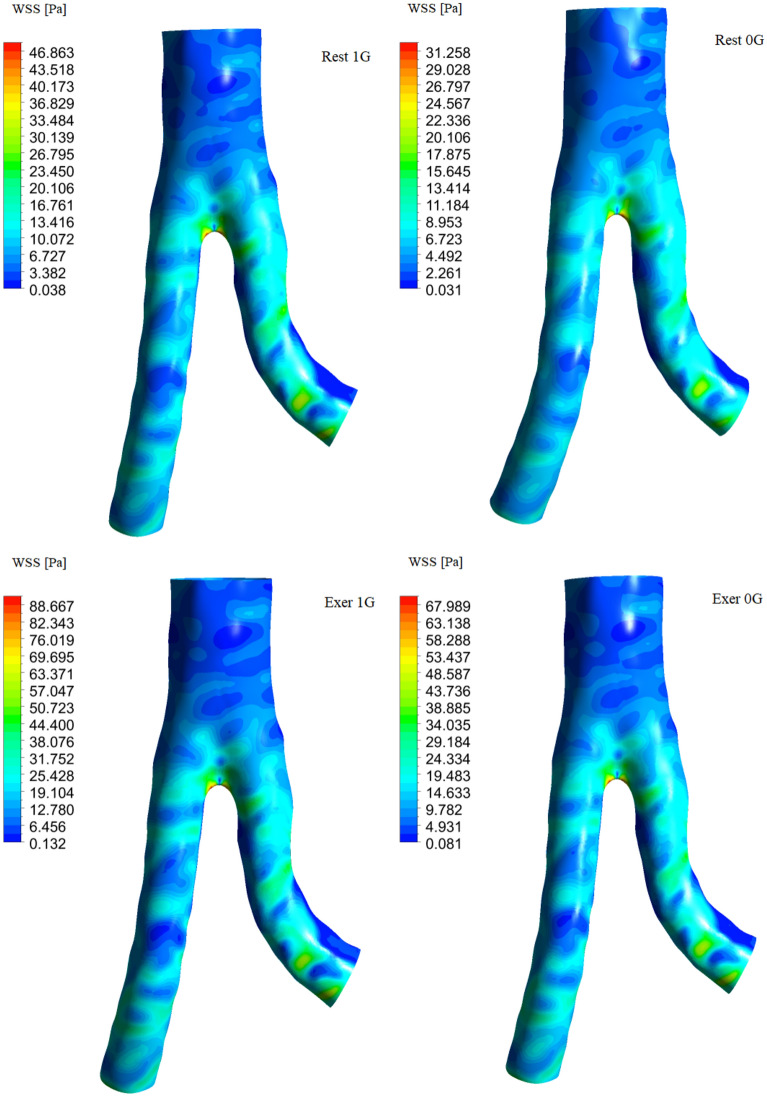
Wall shear stress (WSS) distribution on aortic wall for systolic pressure peak ($t = 0.15 \;{\text{s}}$ for exercise and $t = 0.20 \;{\text{s}}$ for rest).

One of the important mechanical parameters during blood flow is wall shear stress (WSS). Aortic surfaces are characterized by low wall shear stresses and strongly oscillatory blood flow and susceptible to the formation of atherosclerosis^68–70^. The WSS value reflects the friction force between the blood and the walls of blood vessels and may be important in the context of the development and progression of atherosclerosis and other cardiovascular diseases^71^. In Fig. 7, we present the WSS for systolic pressure peak ($t = 0.15 \;{\text{s}}$ for exercise and $t = 0.20 \;{\text{s}}$ for rest). The highest WSS values were observed in the bifurcation region, whereas very low WSS values were observed in the internal curvatures of highly curved vessels (see LIA). This indicates that blood flow does not exert tangential stress on the wall owing to the presence of low-velocity regions. During moderate exercise, we obtained twice the WSS at rest. A microgravity environment reduces the WSS maximum both at rest (by approximately 33%) and during moderate exercise (by approximately 23%).

Local high WSS values are associated with the curvature of arteries in the bifurcation region, meaning that the range of WSS values depends on the specific geometry of the abdominal aorta. Alishahi et al.^62^ demonstrated that WSS can reach 200 Pa, attributed to significant narrowing in the bifurcation region, whereas Bonert et al.^72^ reported a maximum WSS on the order of 40 Pa for an aorta characterized by a wide width in the bifurcation region. For a typical aortic structure, WSS typically ranges from 10 to 70 Pa^73^.

Average WSS values provide more realistic information. Under Earth’s gravity, we observe a mean WSS of 14.77 Pa during moderate exercise and 7.98 Pa at rest. In the case of weightlessness, the corresponding values are 11.37 Pa and 5.28 Pa, resulting in mean WSS changes of 23% during exercise and 34% at rest.

Long-term centrifugal stress on the aortic surface can damage the aorta and lead to aortic aneurysm formation. Due to increase in blood viscosity in the blood vessels, the friction between the aortic endothelium and blood increases, resulting in longer blood flow time in microgravity in the examined section of the vessel.

### Time-averaged wall shear stress

For the systolic pressure peak, was noticed a high velocity of blood flow, WSS values were observed with a high slope that dropped sharply for the diastolic phase of the cardiac cycle; therefore, the time-averaged wall shear stress (TAWSS) and oscillatory shear index (OSI) are significant. The wall shear stress averaged over time is calculated using the formula^74^:

$$TAWSS = \frac{1}{T}\mathop \smallint \limits_{o}^{T} \left| {\tau_{w} } \right|dt$$

where $\tau_{w}$ is the vector of wall shear stress and *T* is the pulsation period. The oscillatory shear index is expressed as^68,75^.

$$OSI = \frac{1}{2}\left( {1 - \frac{{\frac{1}{T}\left| {\mathop \smallint \nolimits_{o}^{T} \tau_{w} dt} \right|}}{TAWSS}} \right)$$

**Figure 8:**
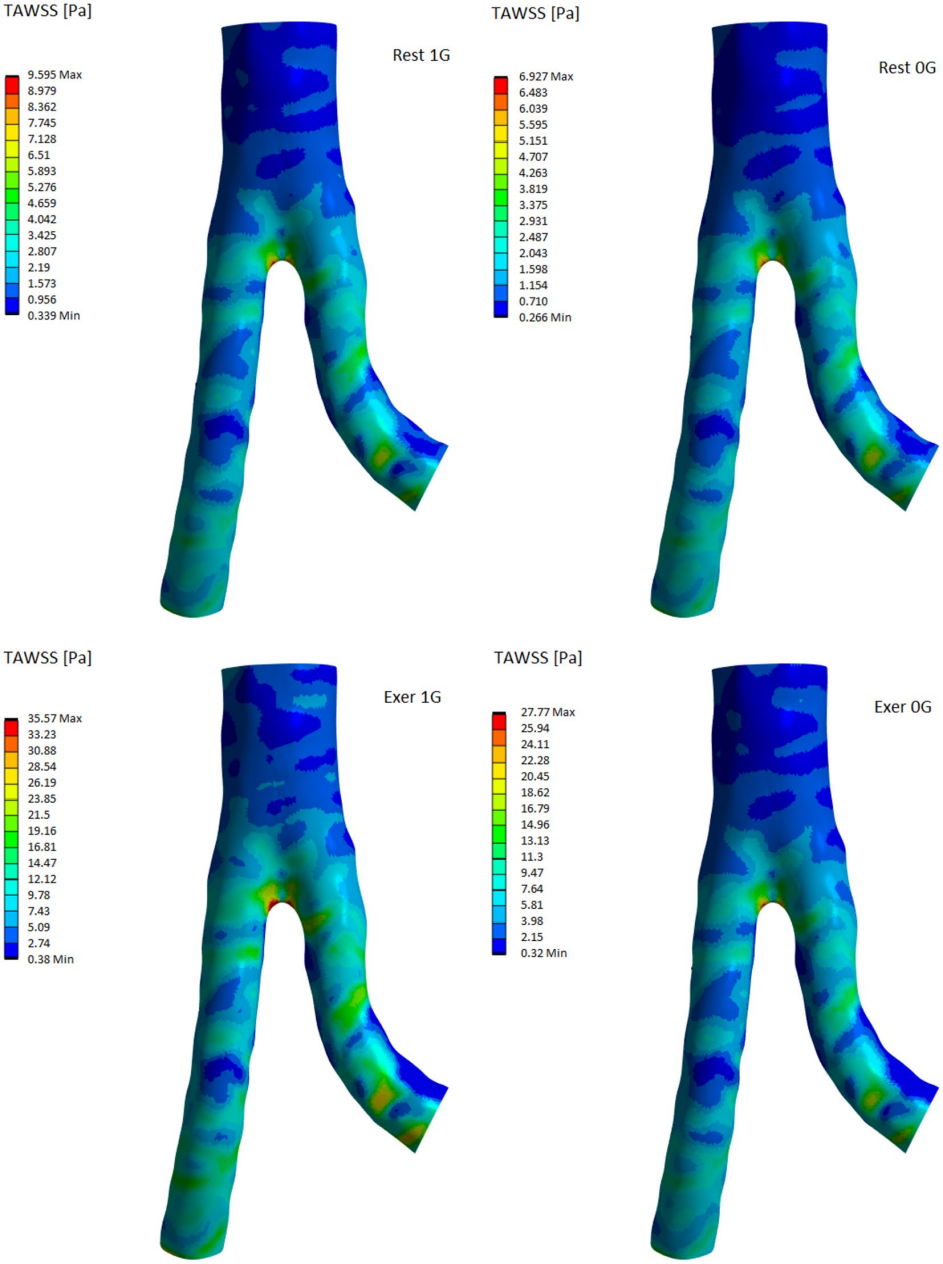
Comparison of TAWSS distribution in the abdominal aorta under 1G (left column) and 0G (right column). The top row corresponds to the results obtained for rest, the bottom row for exercise.

Figure 8 illustrates the distribution of TAWSS in the abdominal aortic wall. The highest TAWSS values were obtained in the bifurcation region and LIA curvature, whereas lower values were obtained in the inner LIA curvature. For the rest of the maximum TAWSS not exceeding the value of 10 Pa, but during moderate exercise, the maximum TAWSS value in the bifurcation region was very high (approximately 35.6 Pa). However, it was on a large surface of the aortic wall, which receives values greater than 10 Pa. Therefore, this situation limits the accumulation of atherosclerotic plaques. In microgravity, the TAWSS value was reduced by approximately 27.8% at rest and 21.9% during moderate exercise. Despite the reduction in TAWSS caused by microgravity, the values obtained for the Exer 0G case were much higher than those for the 1G rest, so moderate exercise can protect against unfavorable hemodynamic parameters of the circulatory system.

A value of approximately 35.6 Pa may indicate the presence of moderate shear stress in the examined abdominal aorta. This indicates that certain stresses may be important for the health of the vessel when assessing hemodynamics, that is, analyzing blood flow and its effect on the vessel. High shear stress on the vessel wall can affect the wall structure and function of the abdominal aorta.

High TAWSS values are associated with arterial geometry changes, such as curvature alterations or aortic bifurcation. For the analysed models, average TAWSS values can also be calculated, providing a clear illustration of WSS distribution on vessel walls. Under Earth’s gravitational acceleration, these values are 5.90 Pa during exercise and 1.85 Pa at rest. In microgravity, the values are 4.68 Pa and 1.33 Pa, respectively, representing a change of 21% during exercise and 28% at rest.

**Figure 9:**
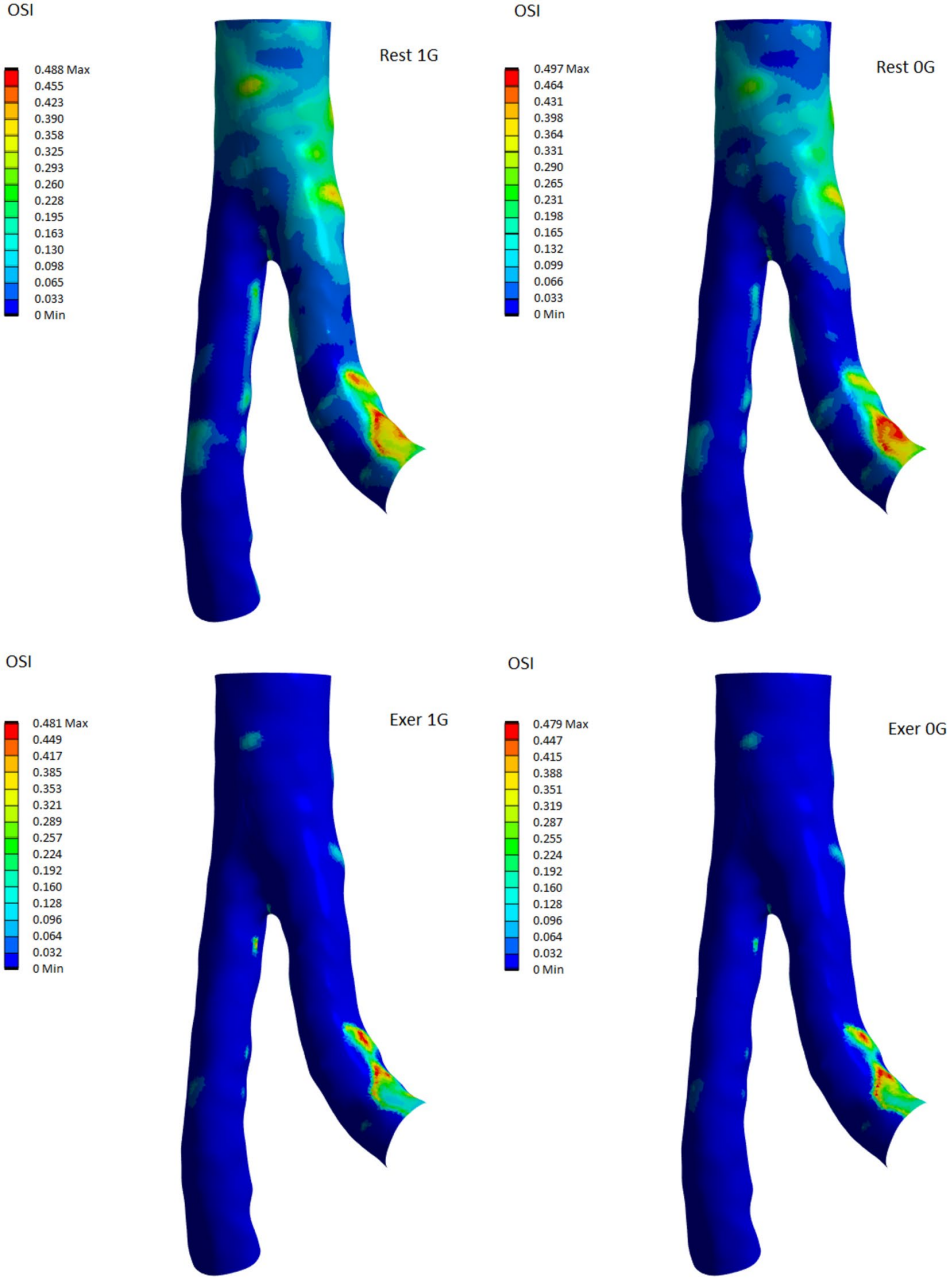
Comparison of OSI distribution in the abdominal aorta under 1G (left column) and 0G (right column). The top row corresponds to the results obtained for rest, the bottom row for exercise.

The OSI is used in connected hemodynamic and can influence the effect of blood on blood vessels and the potential risk of specific pathologies^49^. In hemodynamic studies, significance follows the release of an OSI report, with a value of 0 corresponding to no oscillations and 0.5 corresponding to strong flow oscillations. Figure 9 illustrates the distribution of the oscillatory shear index in the abdominal aorta at rest and during moderate exercise. The maximum OSI values were obtained at the internal curvature of the LIA, that is, in the specifications typical of TAWSS values. At rest, a high OSI occurs on the outer planes in the region of the aortic bifurcation, whereas for moderate exercise in this region, we have small values of OSI. This may be because the flow rate for the diastolic phase is much higher than that for the resting phase, which does not cause oscillations in this device.

The maximum OSI values for rest are higher than for moderate exercise. A similar relationship can be observed for the average OSI values, which for the rest are 0.076 for the 1G case and 0.063 for the 0G case. They are comparable to the results obtained by Caddy et al.^76^. During moderate exercises, we get 0.013 (1G case) and 0.014 (0G case).

Microgravity does not radically change OSI distribution at rest or during moderate exercise^76,77^. A healthy abdominal aorta is typically indicated by an OSI value of zero, which occurs in the primary oscillating blood flow. An OSI value of 0.5 is usually fed with an unstable flow or an oscillation occurring in the flow.

## Discussion

Microgravity can affect hemodynamic, that is, blood flow in the body. Accordingly, a decrease in maximum blood flow velocity was observed at approximately 22.3% during rest and 16.53% during physical exercise. However, the streamlined layout remains unchanged. Associated turbulence was observed in the left iliac artery with large vessel curvature.

A decrease in maximum blood flow velocity may suggest reduced perfusion of tissues and organs, resulting in negative health consequences. Reduced variability in blood flow velocity can affect blood pressure regulation and the hemodynamic balance of the body. In addition, the presence of turbulence in the left iliac artery may indicate blood flow disorders, which may affect microcirculation and the availability of oxygen and nutrients to the tissues. Such changes in arterial hemodynamics may be associated with the risk of cardiovascular complications such as embolism, atherosclerosis, and hypertension.

The analysis of the average velocity magnitude waves indicates microgravity conditions show less variability in speed compared to Earth’s gravity. The maximum speed decreased, whereas the minimum speed increased. In the left iliac artery, higher velocity values were observed during the peak systole phase (systole) and lower values during the early diastole phase (diastole).

Upon exiting microgravity, a sudden decrease in blood velocity in the abdominal aorta is observed under microgravity conditions, especially during systolic pressure. This decrease was smaller during diastolic pressure. Fu et al.^46^, based on repeated beat-to-beat blood pressure measurements, reported a significant reduction in systolic pressure and a very slight reduction in diastolic pressure. Using boundary conditions derived from the reduction in systolic pressure, we determined that the average blood flow rate in microgravity decreases by approximately 16% at rest and by approximately 14.8% during moderate exercise. The mass flow reduction value we obtained is higher than the results reported by Gallo et al.^67^, who observed a 6% reduction in mass flow for the abdominal aorta. This discrepancy arises from the fact that in Gallo et al.^67^, a greater reduction in diastolic pressure was considered compared to our analysis.

Microgravity may be one of the most important factors that affects blood flow in the abdominal aorta, especially during systole. The observed risk of occurrence under microgravity conditions can pose a threat to the circulatory system and requires an appropriate action profile, such as exercise.

The exercises performed increase the pressure and speed of blood flow, which in turn affects the values of wall shear stresses. The TAWSS values we obtained in the presence of Earth’s gravity range up to 35.6 Pa during moderate exercise and 9.6 Pa at rest. Similar values were reported by Taylor et al.^78^ at rest and during exercise. Alimohammadi et al.^79^ reported a TAWSS range of 0–5 Pa at rest.

The state of weightlessness causes a decrease in flow velocity and, consequently, a decrease in the TAWSS value. In our results, this decrease is approximately 22% during exercise and 28% at rest for the mean TAWSS. Sucosky et al.^77^ reported an average change in TAWSS of 22% for the common carotid artery. An important finding is that performing exercises in a state of weightlessness results in much higher values of wall shear stress compared to those during rest in the presence of Earth’s gravity.

Low OSI values indicate stable blood flow, while a value of 0.5 is associated with unstable flow or significant oscillations. Lower OSI values close to zero were expected in healthy abdominal aortas. Proper blood flow in the abdominal aorta is a key process that guarantees the proper functioning and life of a person. The abdominal aorta is the main artery supplying blood from the heart to all systems and parts of the body. Blood flow in the abdominal aorta depends on many factors, such as arterial pressure, blood flow velocity, volume of body water spaces (including the intravascular space), efficiency of the heart muscle, and blood viscosity (dependent on blood morphotic values or serum osmolality).

The countermeasures^80^ aim to reduce the impact of microgravity on the human body, including, among others, daily physical exercises and rehabilitation. Long-term stay in space does not cause negative effects on the human body provided appropriate prevention measures are used, such as the appropriate type and intensity of physical exercise and fluid resuscitation after returning to Earth. Aerobic exercises^81^ are a key countermeasure for physiological changes occurring in the body. Astronauts who take preventive measures against cardiovascular disorders do not experience orthostatic hypotension in the first 24 h after landing, and changes in systolic blood pressure are only transient and return to normal levels after returning to Earth.

Using the Ansys program^82^, simulations can be conducted for a catheter in a blood vessel or simulations^83^ for an intratumoral vessel. Testing blood flow in astronauts’ veins using ANSYS Fluent can provide valuable information on the adaptation of the body to space conditions, potential health threats, and optimization of prevention and medical care in space. The results of this study can be used to develop strategies to counteract the effects of microgravity on astronaut health and to develop new diagnostic and therapeutic tools in space medicine.

In numerical studies of blood flow in the abdominal aorta using the ANSYS Fluent program^84–86^ the results can be analyzed in terms of many variables such as pressure, velocity, shear forces, stresses, and mass flows. These results may clarify the dynamics of blood flow in the abdominal aorta and diagnose possible hemodynamic disorders that may lead to complications, such as atherosclerosis or abdominal aortic aneurysm. Research conducted using ANSYS Fluent can provide valuable information for doctors and engineers dealing with problems related to blood circulation in the body.

### Limitations

The results presented are line up with certain limitations. While we utilized real geometry in our work, it’s important to note that, the same geometry was used in all cases considered. In instances of prolonged exposure to microgravity, changes in body size are observed, potentially leading to deformation of the abdominal aorta. A more comprehensive analysis would necessitate the utilization of geometry derived from computed tomography scans conducted on astronauts before and after space missions, as the effects of microgravity may persist.

Furthermore, our analysis was conducted under the assumption of rigid walls. However, it is possible to incorporate fluid and vessel wall motion simulations using Fluid–Structure Interaction (FSI) technology.

Validation of our model was achieved solely through comparison with existing literature data. Ideally, a comparison with actual measurements would provide a more robust validation. However, conducting measurements under microgravity conditions presents practical challenges.

## Data Availability

The datasets used and/or analyzed during the current study available from the corresponding author on reasonable request.
